# Supplementary material for: Condensation of 4-Tert-butyl-2,6-dimethylbenzenesulfonamide with Glyoxal and Reaction Features: A New Process for Symmetric and Asymmetric Aromatic Sulfones
Source: Molecules. 2022 Nov 12;27(22):7793. doi: 10.3390/molecules27227793 (PMC9697454; doi:10.3390/molecules27227793)
Supplement: Supplementary file 1 [file molecules-27-07793-s001.zip › molecules-1993142-supplementary.pdf]

Supplementary Materials

Condensation of 4-tert-butyl-2,6-dimethylbenzenesulfonamide with glyoxal and reaction features. A new process for symmetric and asymmetric aromatic sulfones

Artyom E. Paromov *, Sergey V. Sysolyatin and Irina A. Shchurova

Laboratory for Chemistry of Nitrogen Compounds, Institute for Problems of Chemical and Energetic Technologies, Siberian Branch of the Russian Academy of Sciences (IPCET SB RAS), Biysk 659322, Russia
*Correspondence: nitrochemistry@mail.ru

Table of contents

^{1}H and ^{13}C NMR of compound **2** S2

^{1}H and ^{13}C NMR of compound **3** S4

^{1}H and ^{13}C NMR of compound **4** S6

^{1}H and ^{13}C NMR of compound **6(H+)** S8

^{1}H and ^{13}C NMR of compound **9** S10

^{1}H and ^{13}C NMR of compound **12** S12

^{1}H and ^{13}C NMR of compound **13** S14

^{1}H and ^{13}C NMR of compound **14** S16

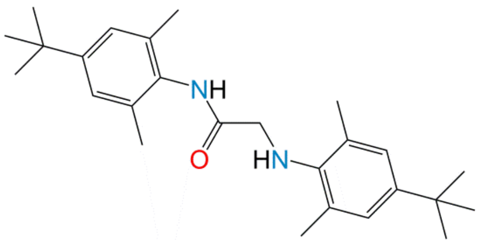

2-((4-(Tert-butyl)-2,6-dimethylphenyl)sulfonamido)-N-((4-(tert-butyl)-2,6-dimethylphenyl)sulfonyl)-acetamide (**2**)

^{1}H (400 MHz) NMR ($CDCl_3$, 24°C)

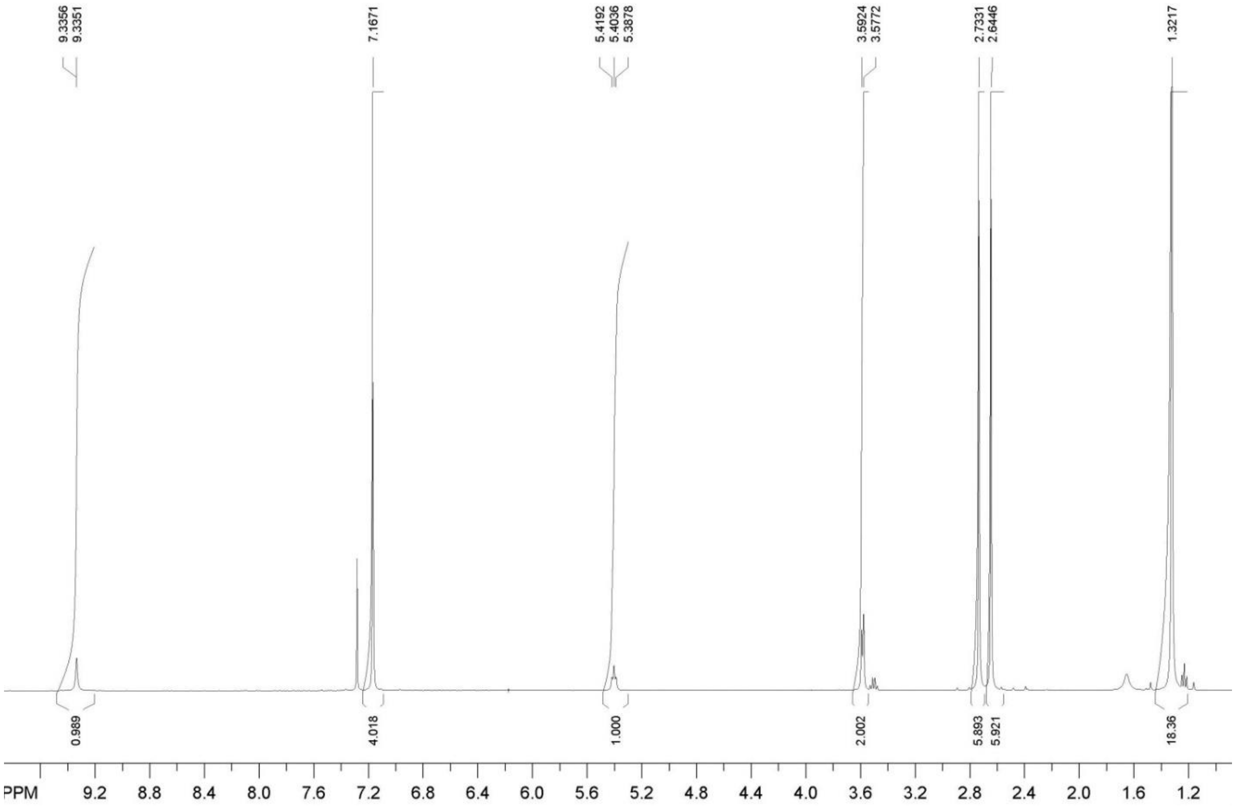

Figure S1. ^{1}H NMR ($CDCl_3$) spectrum of compound **2**

^{13}C (100 MHz) NMR ($CDCl_3$, 24°C)

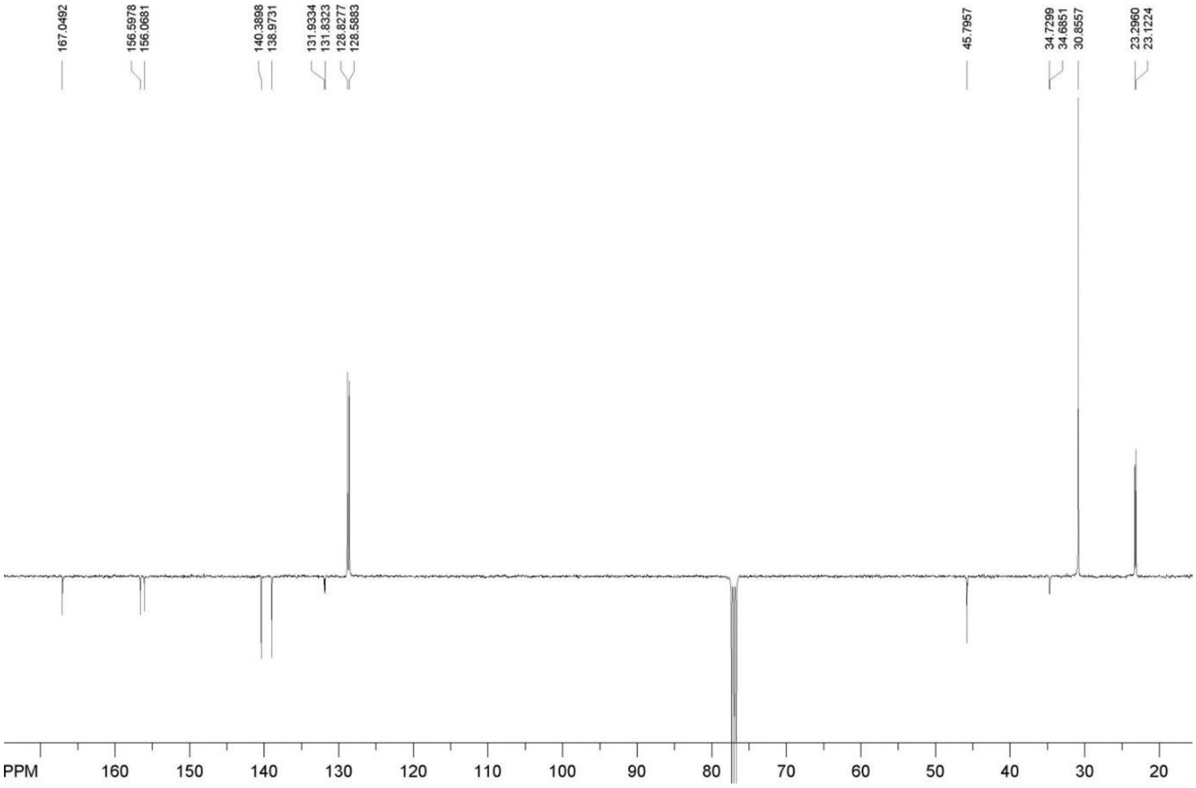

Figure S2. ^{13}C NMR ($CDCl_3$) spectrum of compound **2**

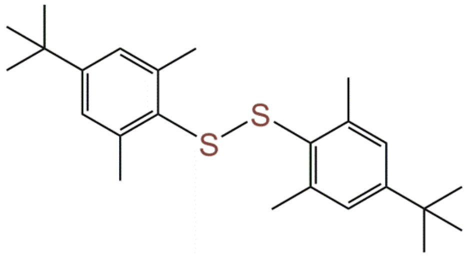

1,2-Bis(4-(tert-butyl)-2,6-dimethylphenyl)disulfane (**3**)

^{1}H (400 MHz) NMR ($CDCl_3$, 24°C)

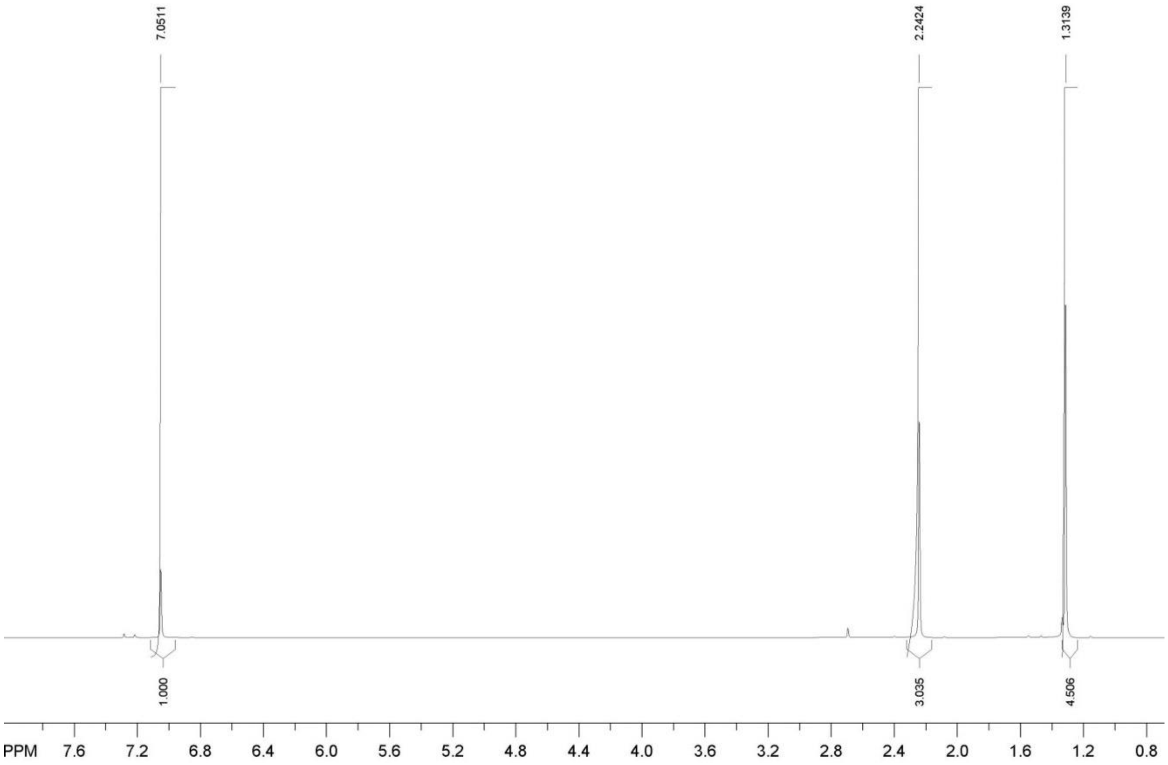

Figure S3. ^{1}H NMR ($CDCl_3$) spectrum of compound **3**

^{13}C (100 MHz) NMR ($CDCl_3$, 24°C)

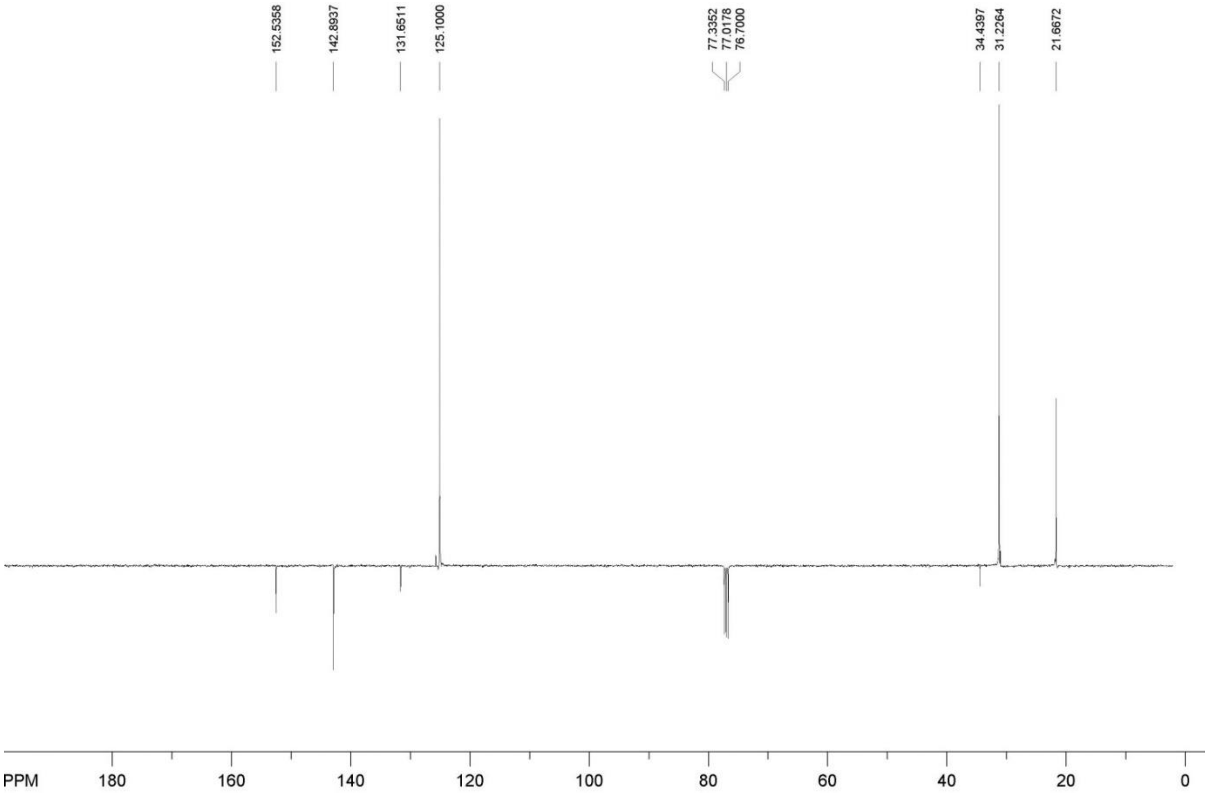

Figure S4. ^{13}C NMR ($CDCl_3$) spectrum of compound **3**

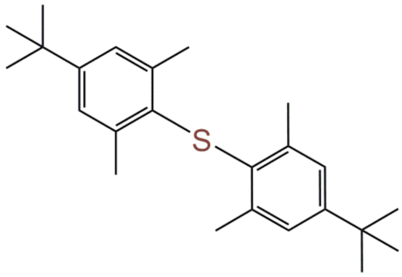

Bis(4-(tert-butyl)-2,6-dimethylphenyl)sulfane (**4**)

^{1}H (400 MHz) NMR (acetone-d, 24°C)

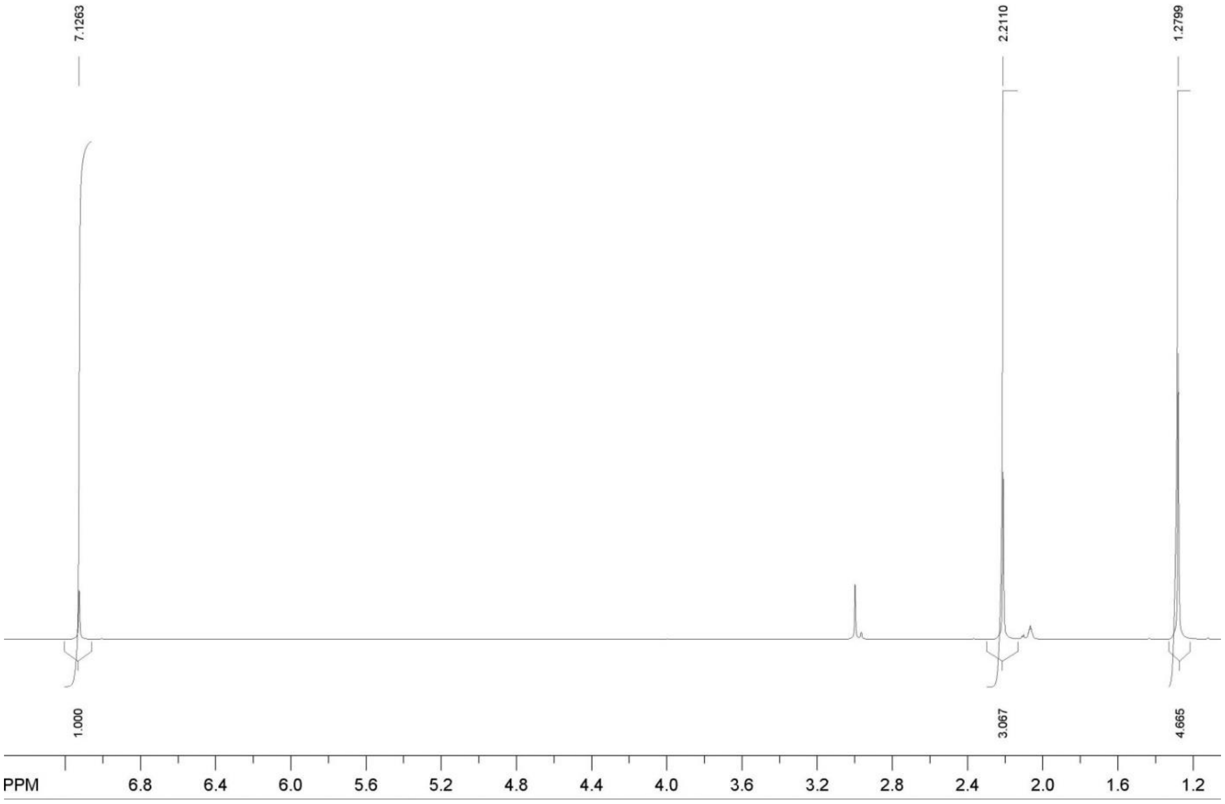

Figure S5. ^{1}H NMR (acetone-d) spectrum of compound **4**

^{13}C (100 MHz) NMR (acetone-d, 24°C)

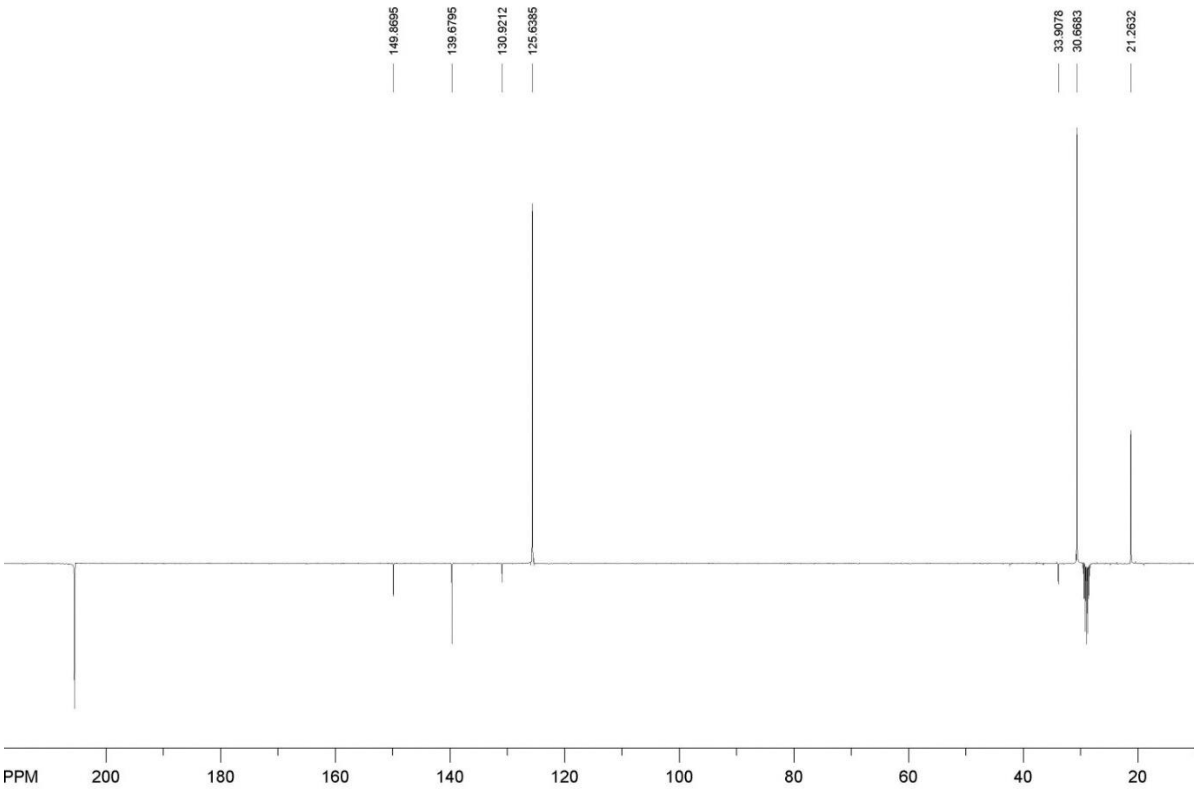

Figure S6. ^{13}C NMR (acetone-d) spectrum of compound **4**

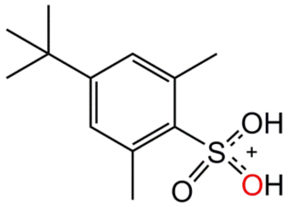

Protonated 4-(tert-butyl)-2,6-dimethylbenzenesulfonic acid (**6(H⁺)**)

^{1}H (400 MHz) NMR (CDCl$_3$, 24°C)

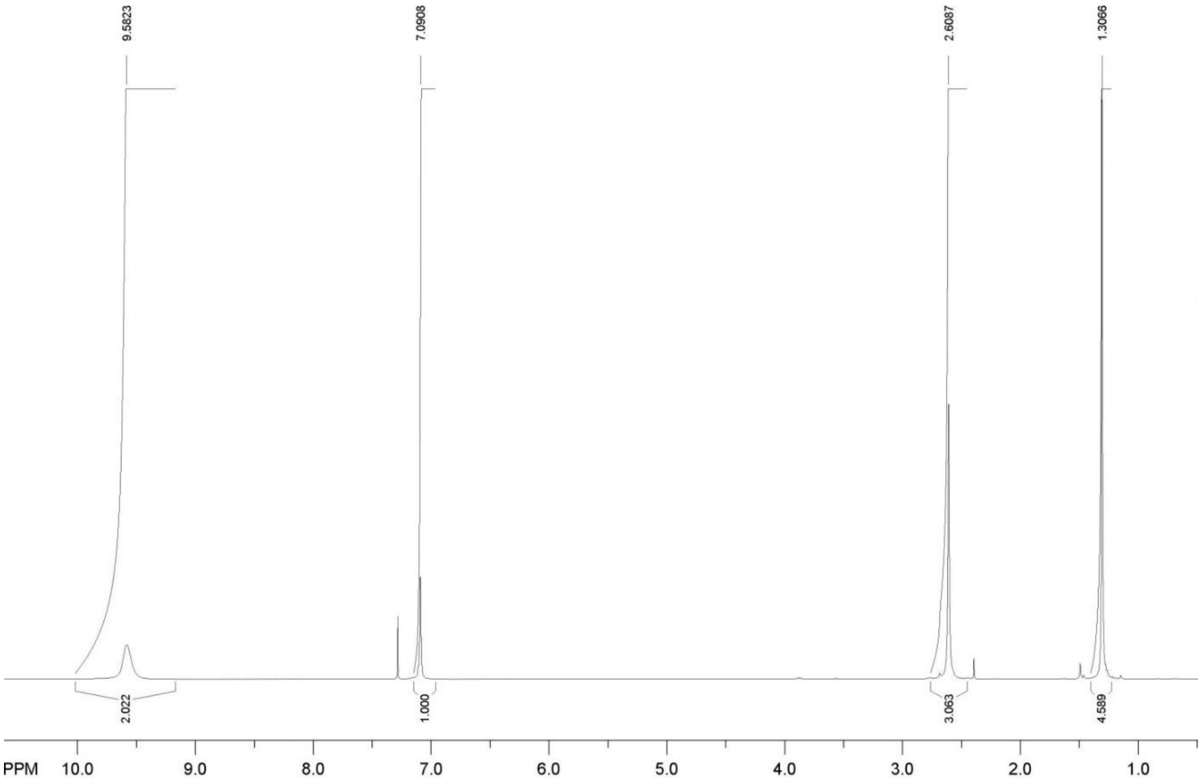

Figure S7. ^{1}H NMR (CDCl$_3$) spectrum of compound **6(H$^+$)**

^{13}C (100 MHz) NMR ($CDCl_3$, 24^oC)

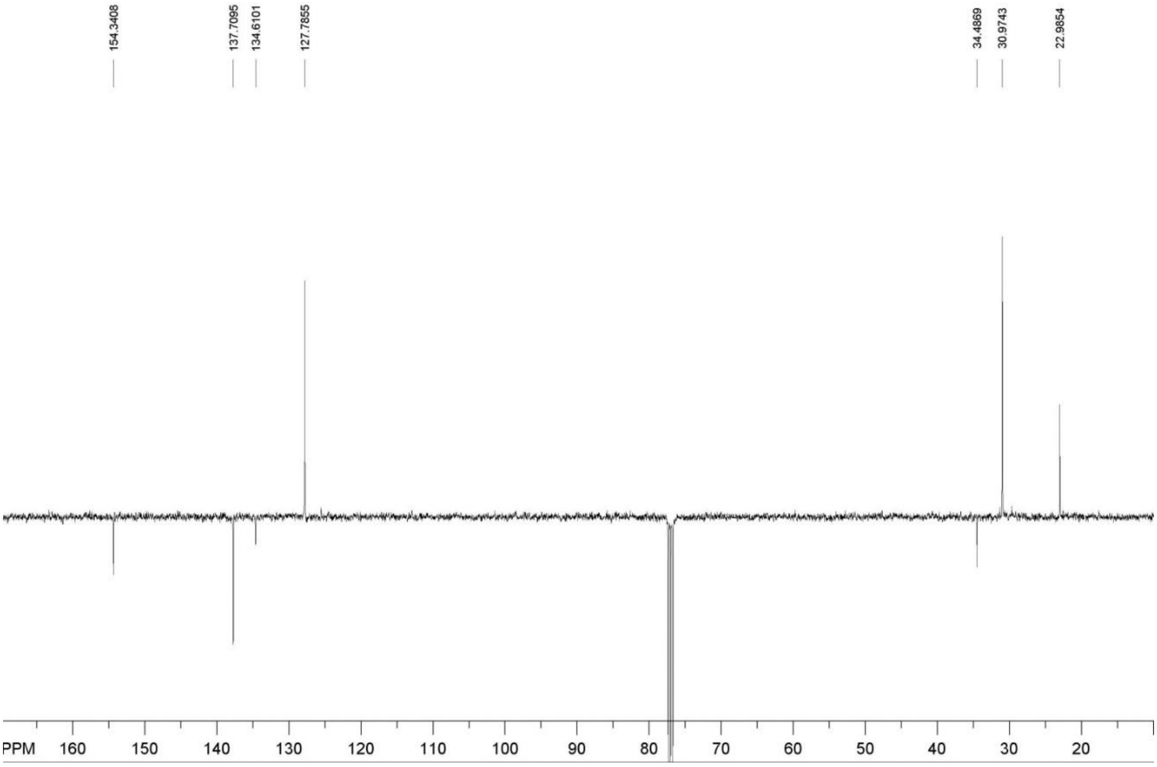

Figure S8. ^{13}C NMR ($CDCl_3$) spectrum of compound **6(H$^+$)**

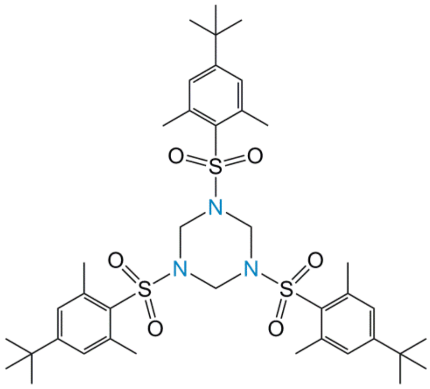

1,3,5-Tris((4-(tert-butyl)-2,6-dimethylphenyl)sulfonyl)-1,3,5-triazinane (**9**)

^{1}H (400 MHz) NMR ($CDCl_3$, 24°C)

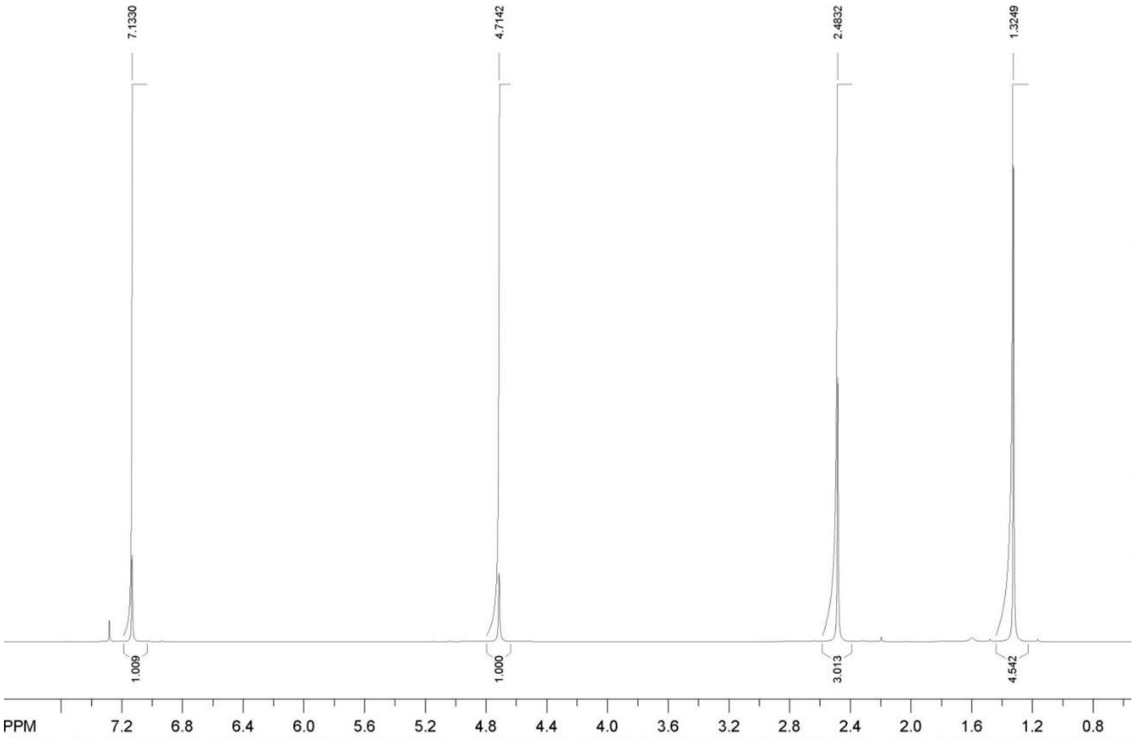

Figure S9. ^{1}H NMR ($CDCl_3$) spectrum of compound **9**

^{13}C (100 MHz) NMR ($CDCl_3$, 24°C)

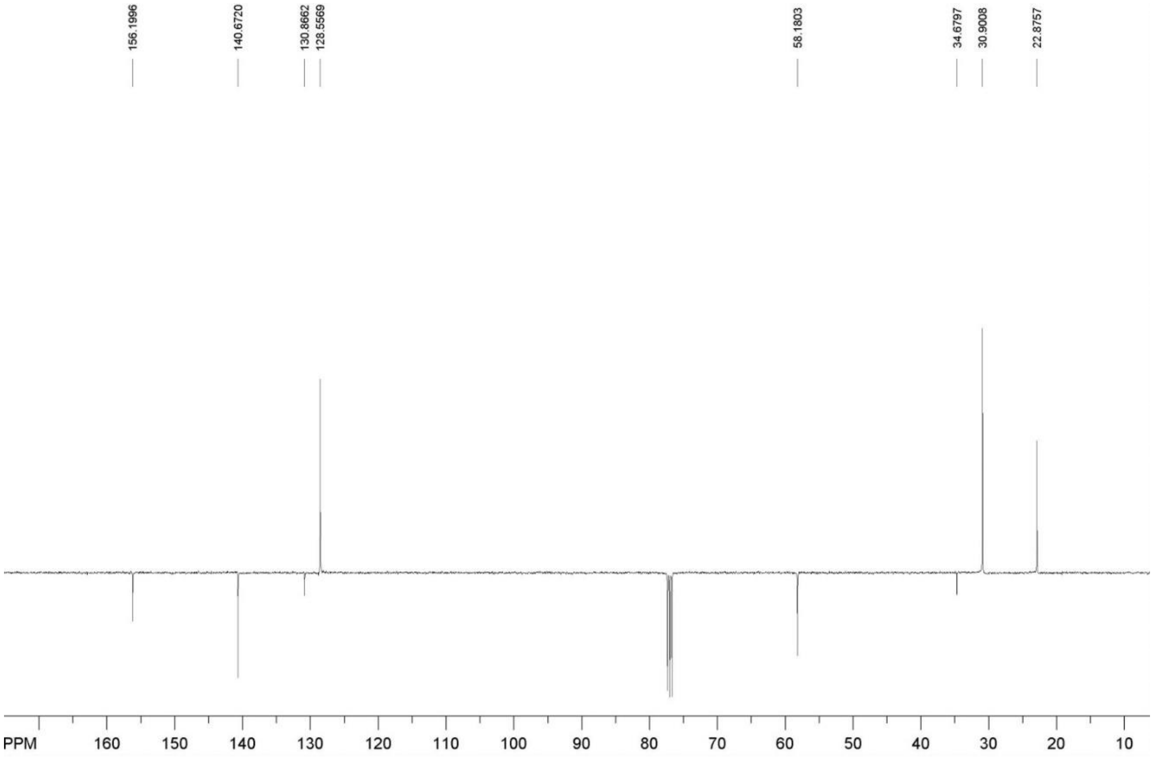

Figure S10. ^{13}C NMR ($CDCl_3$) spectrum of compound **9**

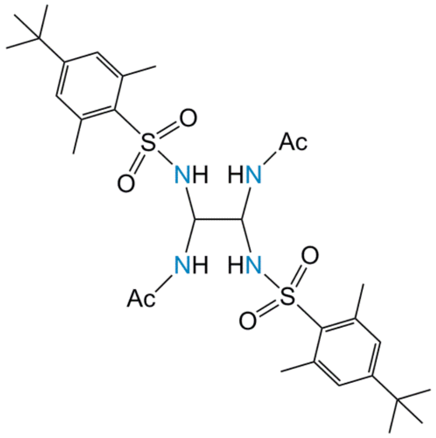

N,N'-(1,2-Bis((4-(tert-butyl)-2,6-dimethylphenyl)sulfonamido)ethane- 1,2-diyl)diacetamide (**12**)

^{1}H (400 MHz) NMR (DMSO-d6, 24°C)

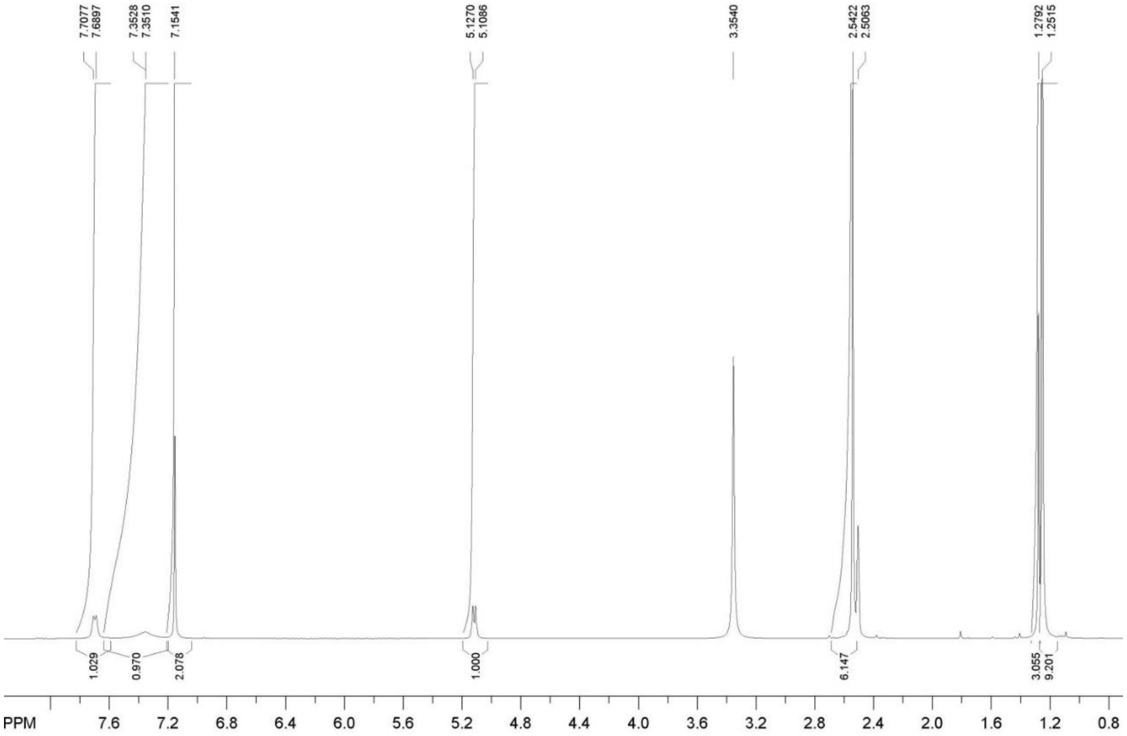

Figure S11. ^{1}H NMR (DMSO-d6) spectrum of compound **12**

^{13}C (100 MHz) NMR (DMSO-d6, 24°C)

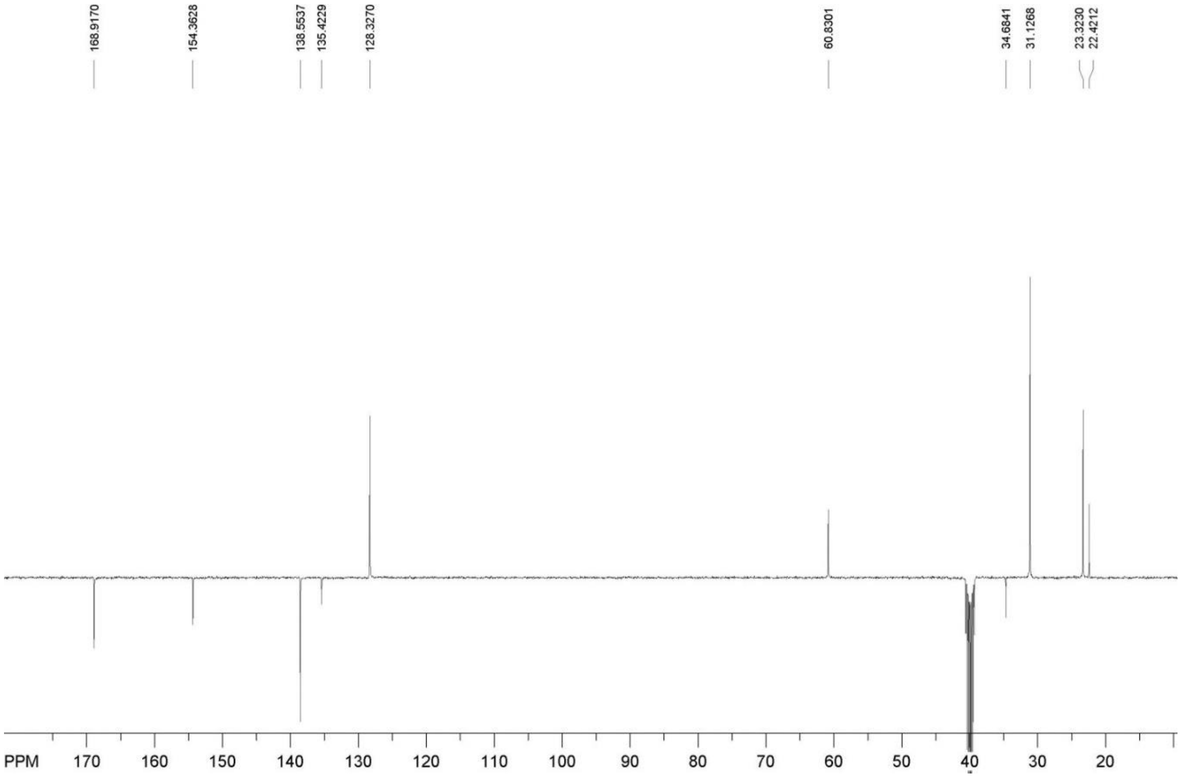

Figure S12. ^{13}C NMR (DMSO-d6) spectrum of compound **12**

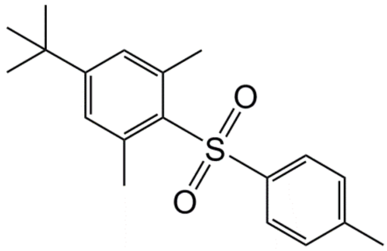

5-(Tert-butyl)-1,3-dimethyl-2-tosylbenzene (**13**)

^{1}H (400 MHz) NMR ($CDCl_3$, 24°C)

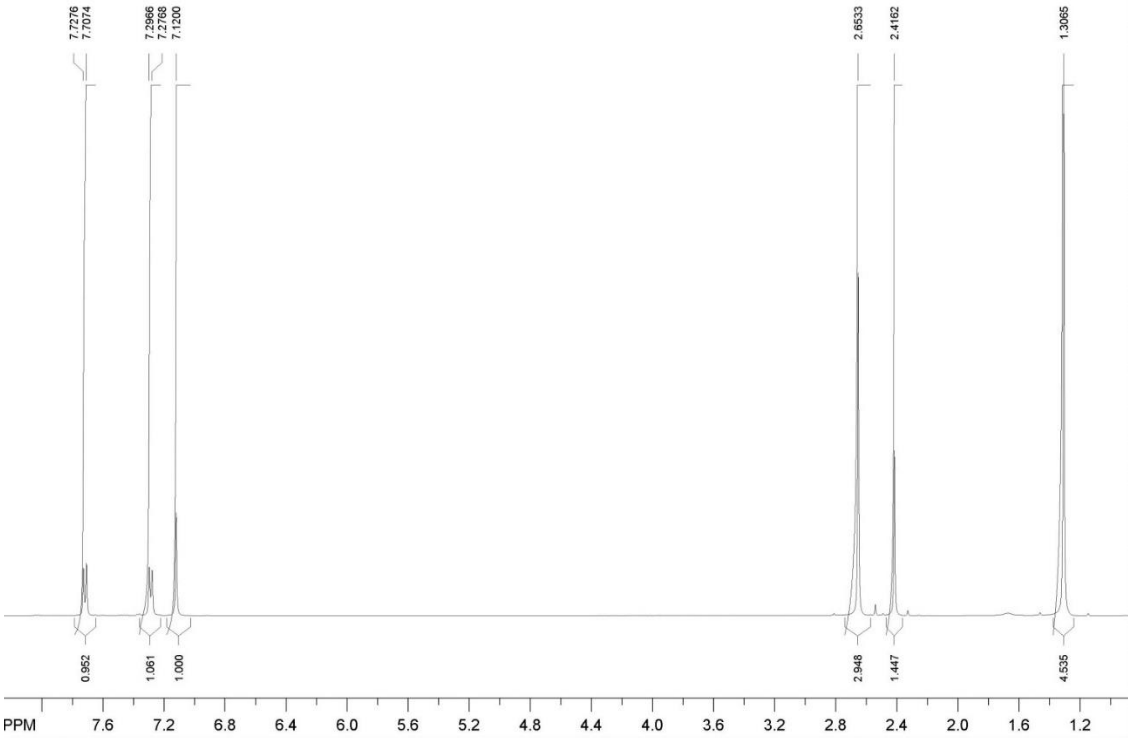

Figure S13. ^{1}H NMR ($CDCl_3$) spectrum of compound **13**

^{13}C (100 MHz) NMR ($CDCl_3$, 24°C)

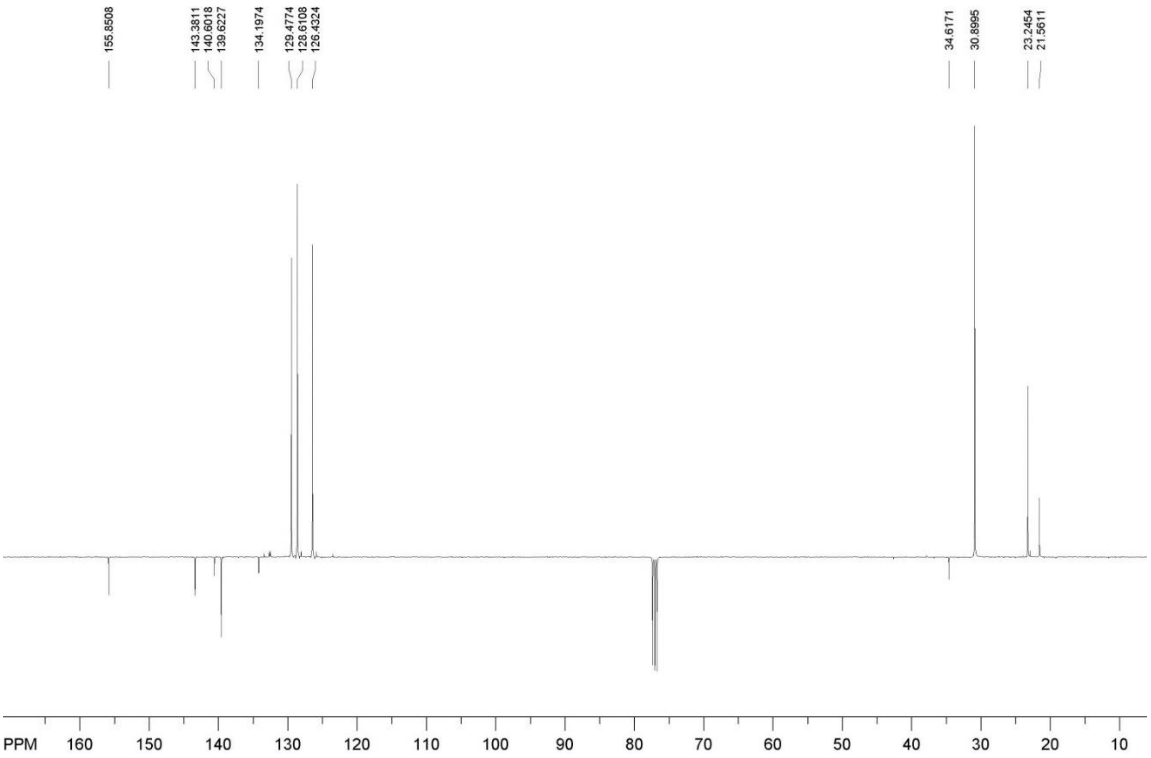

Figure S14. ^{13}C NMR ($CDCl_3$) spectrum of compound **13**

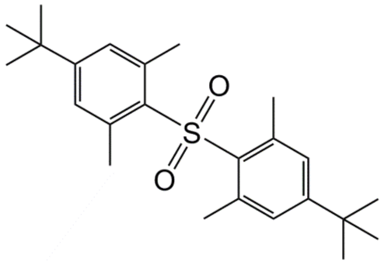

2,2'-Sulfonylbis(5-(tert-butyl)-1,3-dimethylbenzene) (**14**)

^{1}H (400 MHz) NMR (DMSO-d6, 24°C)

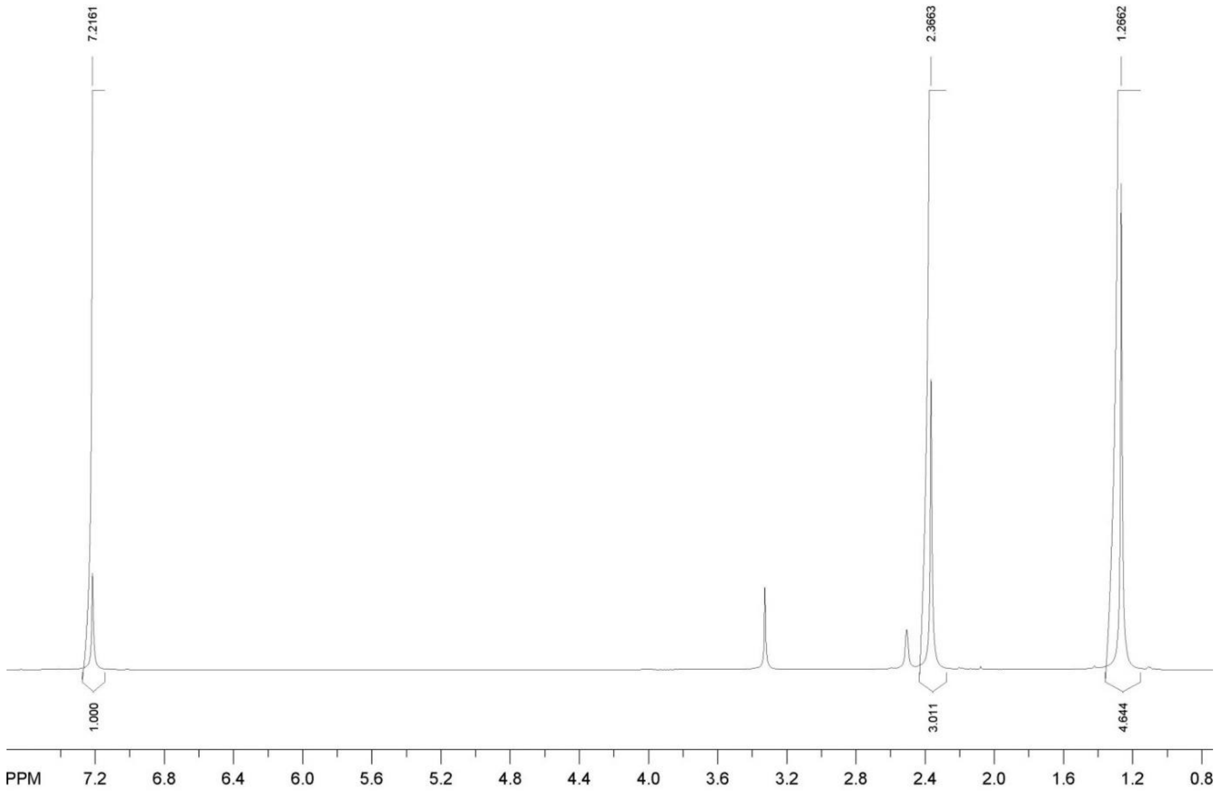

Figure S15. ^{1}H NMR (DMSO-d6) spectrum of compound **14**

^{13}C (100 MHz) NMR (DMSO-d6, 24°C)

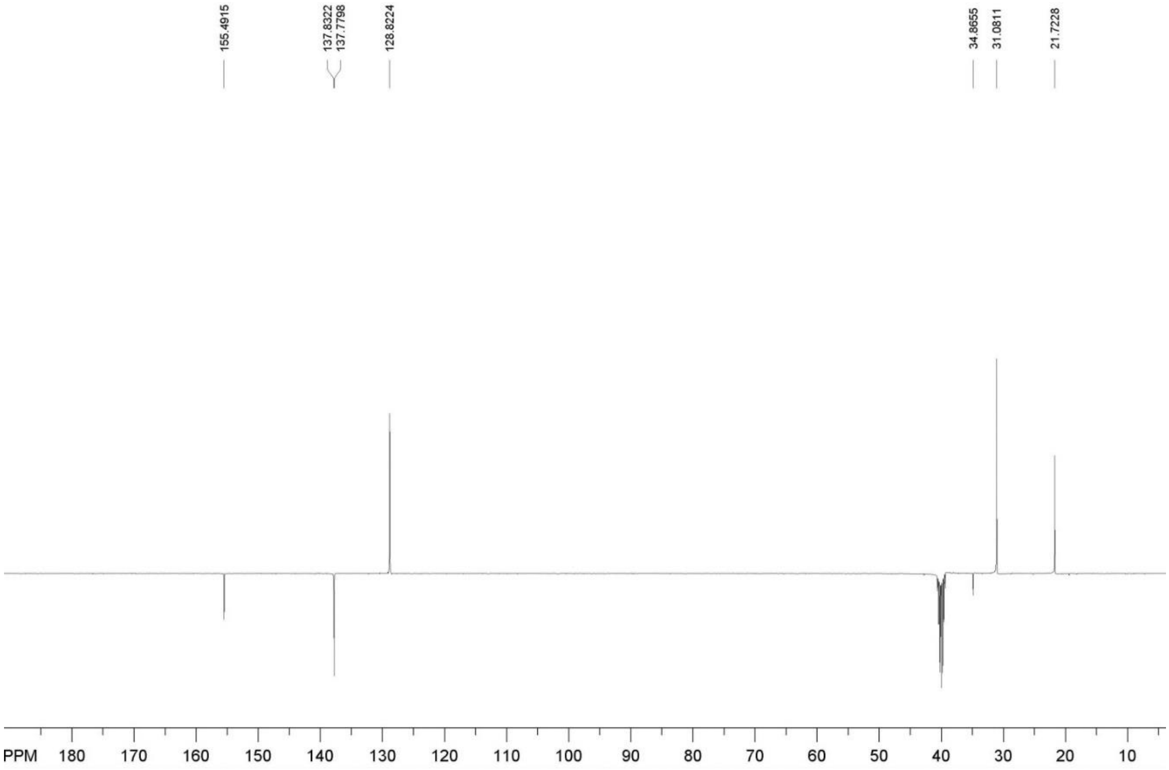

Figure S16. ^{13}C NMR (DMSO-d6) spectrum of compound **14**
